# Unraveling the mystery of white matter in depression: A translational perspective on recent advances

**DOI:** 10.1002/brb3.2629

**Published:** 2022-06-01

**Authors:** Mate Abraham, Annakarina Mundorf, Katja Brodmann, Nadja Freund

**Affiliations:** ^1^ Division of Experimental and Molecular Psychiatry Department of Psychiatry Psychotherapy and Preventive Medicine LWL University Hospital Ruhr‐University Bochum Bochum Germany; ^2^ Institute for Systems Medicine and Department of Human Medicine MSH Medical School Hamburg Hamburg Germany; ^3^ Department of Neuroimaging Institute of Psychiatry, Psychology and Neuroscience, King's College London London UK

**Keywords:** Fractional anisotropy, Major depressive disorder, MRI, postmortem, primates, rodents

## Abstract

**Background:**

Numerous cortical and subcortical structures have been studied extensively concerning alterations of their integrity as well as their neurotransmitters in depression. However, connections between these structures have received considerably less attention.

**Objective:**

This systematic review presents results from recent neuroimaging as well as neuropathologic studies conducted on humans and other mammals. It aims to provide evidence for impaired white matter integrity in individuals expressing a depressive phenotype.

**Methods:**

A systematic database search in accordance with the PRISMA guidelines was conducted to identify imaging and postmortem studies conducted on humans with a diagnosis of major depressive disorder, as well as on rodents and primates subjected to an animal model of depression.

**Results:**

Alterations are especially apparent in frontal gyri, as well as in structures establishing interhemispheric connectivity between frontal regions. Translational neuropathological findings point to alterations in oligodendrocyte density and morphology, as well as to alterations in the expression of genes related to myelin synthesis. An important role of early life adversities in the development of depressive symptoms and white matter alterations across species is thereby revealed. Data indicating that stress can interfere with physiological myelination patterns is presented. Altered myelination is most notably present in regions that are subject to maturation during the developmental stage of exposure to adversities.

**Conclusion:**

Translational studies point to replicable alterations in white matter integrity in subjects suffering from depression across multiple species. Impaired white matter integrity is apparent in imaging as well as neuropathological studies. Future studies should focus on determining to what extent influencing white matter integrity is able to improve symptoms of depression in animals as well as humans.

## Graphical abstract

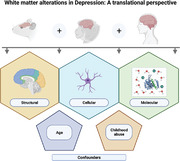

## 1 INTRODUCTION

Major depressive disorder (MDD) is a chronic health condition causing considerable distress to affected patients. Moreover, MDD is one of the leading causes of disability worldwide: according to the Lancet Global Burden of Disease from 2017, depressive disorders (comprising MDD and dysthymia) were the third‐largest contributor to Years Lost to Disability on a global scale, preceded only by low back pain and headache disorders (James et al., 2018). Furthermore, MDD is often associated with other diseases, such as anxiety disorders, type II diabetes, chronic back pain, and rheumatic diseases, thus further highlighting its clinical relevance (Baerwald et al., 2019; Currie & Wang, 2005; Eaton et al., 1996; Fava et al., 2000).

Despite MDD being a large health and socioeconomic burden, to date, there is no universal consensus about its pathogenesis. Numerous studies, both in humans and animals, have been conducted to discover the mechanisms underlying the condition. Even though there has been a lot of progress in this field, the exact cytological correlates of mood disorders are still not clear.

For a long time, it was difficult to determine functional and structural changes in the brains of patients suffering from mental disorders, as these changes are frequently not clearly apparent in postmortem tissue, and in vivo methods were not capable of identifying alterations. However, recent advances of imaging technologies allow for a more sophisticated analysis of structural and functional alterations in neuronal structures of living individuals, thereby revealing significant alterations in psychiatric patients (Zhan & Yu, 2015). One of the most important instruments that have recently gained increasing relevance in investigating brain structures are diffusion tensor imaging metrics, an advancement of magnetic resonance imaging (MRI) technology. While MRI represents a powerful technology to create high‐resolution images of patients’ central nervous systems, diffusion tensor imaging metrics can yield useful additional information about neuronal structures. Until recently, MRI has mostly been used to investigate volumetric aberrations of brain structures in depressed patients. One of the most consistent results in this context is a reduced hippocampus size, as reviewed amongst others by Videbech and Ravnkilde (2004). An emerging aspect of MDD research that has recently gained increasing attention is alterations in white matter (WM). The advancement of diffusion tensor imaging in MRI has allowed for a more profound investigation of WM architecture in humans as well as in rodents. The most common metric used to analyze aberrant diffusivity in WM in MDD is fractional anisotropy (FA), an invaluable tool to analyze nerve fiber density and orientation. As FA exhibits decreased values in healthy individuals under the age of 18, as well as a rapid decline above the age of 65 (Kochunov et al., 2011), the current review only includes MRI studies conducted on individuals aged 18–65. Moreover, antidepressant treatment is able to exhibit both short‐ and long‐term effects on WM microstructure, leading to alterations in FA, radial diffusivity, mean diffusivity, and axial diffusivity (Lai et al., 2013; Seiger et al., 2021; Yoo et al., 2007; Zeng et al., 2012). Therefore, to avoid a history of antidepressant treatment acting as a confounder, the current review only comprises studies that investigated medication‐naïve patients. This approach allows for a more meaningful translational comparison between humans suffering from MDD and alterations identified in animal models of depression, as it highlights WM abnormalities in depression before treatment.

Preclinical studies in rats and mice allow for a disentanglement of neuronal implications on a molecular level using animal models of psychiatric disorders such as addiction (Mundorf et al., 2020), bipolar disorder (Beyer et al., 2021), schizophrenia (Juckel et al., 2021; Mundorf, Kubitza, et al., 2021; Wegrzyn et al., 2021), as well as stress‐induced impairments (Bölükbas et al., 2020; Mundorf et al., 2019; Mundorf, Koch, et al., 2021). Preclinical studies conducted mostly on rats and mice as well as clinical studies investigating humans both play an important role in furthering the understanding of the pathogenesis of MDD. However, only a few reviews have focused on translating findings concerning WM in animal studies to humans and vice versa (Edgar & Sibille, 2012; McNamara & Lotrich, 2012). Recent advances in MRI allow for a closer look at alterations in diffusion metrics in WM in both clinical and preclinical trials. They furthermore provide the valuable possibility of comparing findings in different species, thus allowing for further insight into the mechanisms underlying these alterations.

This systematic review aims to provide an up‐to‐date, concise overview of recent advances concerning WM alterations in humans, primates, and rodents. Therefore, it comprises four categories of studies: (I) Human MRI studies in patients with the clinical diagnosis of MDD, (II) MRI studies conducted on rodents and primates exposed to an animal model of depression, (III) postmortem studies conducted on deceased MDD patients, as well as (IV) postmortem studies conducted on rodents and primates with an animal model of depression. Considering these different approaches will allow for the assessment of cytological alterations associated with findings in MRI studies in all species.

## 2 METHODS

The database PubMed was searched using a Boolean search strategy for each of the four aspects of the study while limiting search results to articles from January 1, 2009 to October 30, 2021. Review articles and studies that did not examine MDD were excluded. Studies from humans, rodents, and primates were included, although results concerning primates are rare. Studies were assessed by two raters independently (MA and AM) and a third independent rater (NF) was consulted in case of discrepancies. The PRISMA checklist for systematic reviews was followed during the preparation of the article. The risk of bias was assessed for each study by the raters independently. Possible risks of bias are indicated in the tables of the Supporting Information alongside each study. In the following, specific criteria for the four aspects of this review are presented. A full list of all included and excluded studies is provided in the tables of the Supporting Information.

### 2.1 MRI studies in patients

**FIGURE 1:**
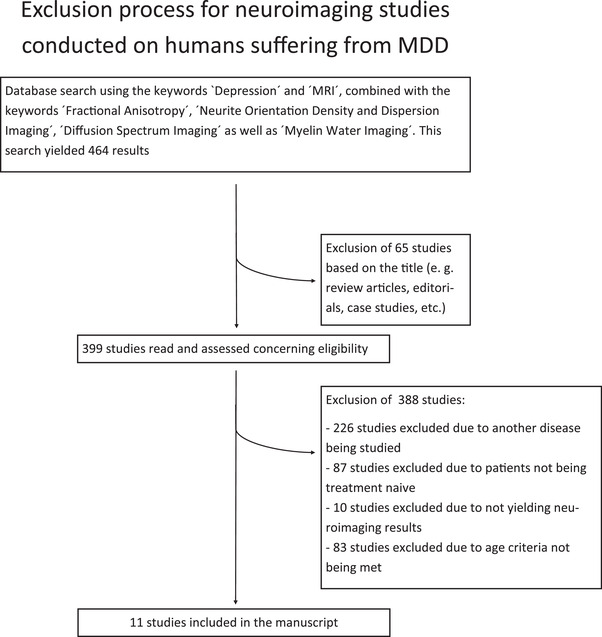
Flowchart depicting the exclusion process for MRI studies conducted in patients

The keywords “Depression” and “MRI” were combined with the keywords “Fractional Anisotropy,” “Neurite Orientation Density and Dispersion Imaging,” “Diffusion Spectrum Imaging,” or “Myelin Water Imaging” successively to search the database PubMed. The search results were then limited to studies conducted on humans. This search yielded 464 studies. Manual selection of these studies was conducted to verify whether publications met any of the exclusion criteria. Exclusion criteria were the following: patients studied have (I) a psychiatric disorder other than MDD OR (II) a diagnosed neurological disorder OR (III) a documented traumatic brain injury OR (IV) do not meet the age criteria (between 18 and 65 years of age) OR (V) have received either antidepressant medication or psychotherapy in their lifetime. This resulted in the inclusion of 11 studies and the exclusion of 453 studies (see Table S1 and Figure 1). All included studies obtained informed consent and were carried out in accordance with the Declaration of Helsinki.

### 2.2 MRI studies in rodents and primates

**FIGURE 2:**
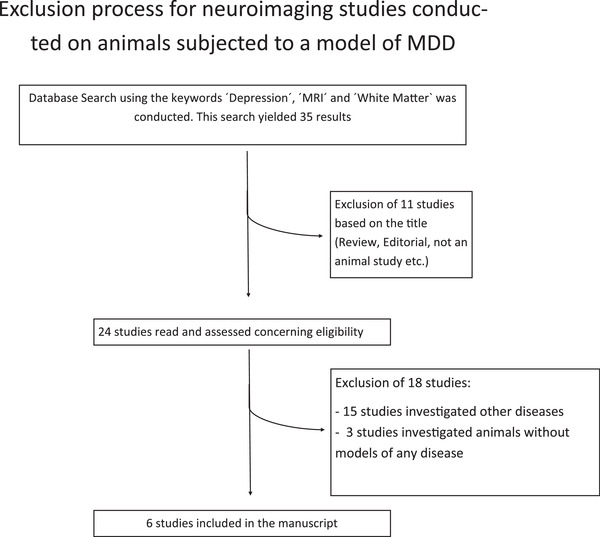
Flowchart depicting the exclusion process for MRI studies conducted in rodents and primates

The keywords “Depression” and “MRI” were combined with the keywords “White Matter,” “Neurite Orientation Density and Dispersion Imaging,” “Diffusion Spectrum Imaging,” as well as “Myelin Water Imaging” to search the database PubMed. Search results were limited to “Other Animals.” This yielded 35 results. Studies were then evaluated concerning exclusion criteria. These were the following: (I) animals studied were other than primates, rats, or mice OR (II) a disease other than MDD was induced in the animal OR (III) the study did not examine WM alterations OR (IV) the animals received antidepressant medication. This resulted in the inclusion of six studies and the exclusion of 29 studies (see Table S2 and Figure 2). All included animal experiments complied with the EU Directive 2010/63/EU for animal experiments, or with comparable guidelines for the ethical treatment of animals in research.

### 2.3 Postmortem studies in patients

**FIGURE 3:**
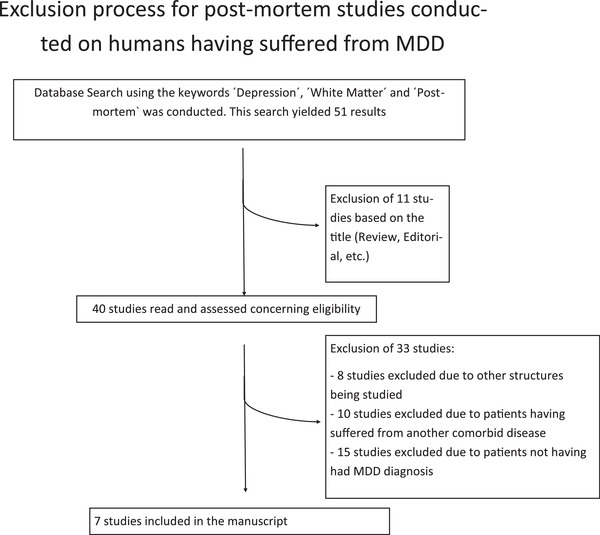
Flowchart depicting the exclusion process for postmortem studies conducted in patients

The keywords “White Matter,” “Depression,” and “Postmortem” were utilized to conduct a PubMed database search. This search yielded 51 studies, which were evaluated concerning exclusion criteria. Exclusion criteria were the following: the patients (I) did not have a diagnosis of MDD OR (II) had a further psychiatric or neurological disorder OR (III) the study did not investigate WM alterations OR (IV) only investigated late‐life depression. This resulted in the inclusion of seven studies and the exclusion of 44 studies (see Table S3 and Figure 3). All included studies obtained informed consent from the closest living relative and were carried out in accordance with the Declaration of Helsinki.

### 2.4 Postmortem studies in primates and rodents

**FIGURE 4:**
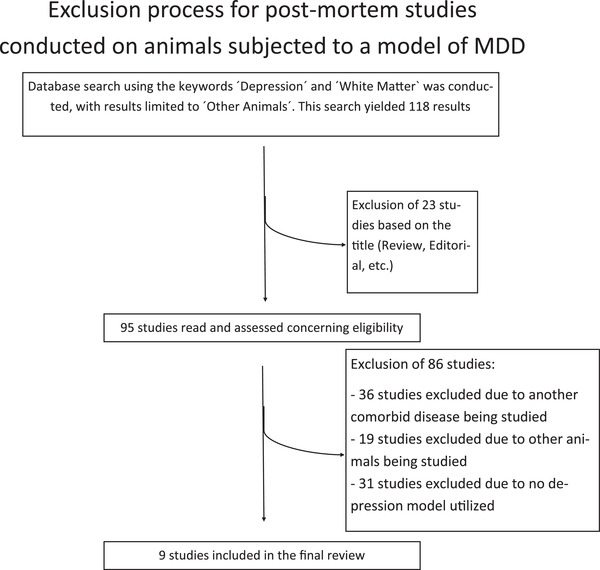
Flowchart depicting the exclusion process for MRI studies conducted in patients

A PubMed database search using the keywords “Depression” and “White Matter” was conducted, while limiting the search results to “Other Animals.” This yielded 118 results, which were then evaluated concerning exclusion criteria. These criteria were the following: (I) animals studied were other than rats, mice, or primates OR (II) a disease other than MDD was induced in the animal OR (III) the study did not examine WM alterations. The application of the exclusion criteria resulted in the inclusion of nine studies and the exclusion of 109 studies (see Table S4 and Figure 4). All included animal experiments complied with the EU Directive 2010/63/EU for animal experiments, or with comparable guidelines for the ethical treatment of animals in research.

## 3 RESULTS OF MRI STUDIES IN HUMANS AND ANIMALS

For an overview of all included MRI studies discussed in this section, confer Tables 1 and 2. In the following, results concerning WM tracts establishing inter‐ and intrahemispheric connectivity are presented.

**TABLE 1:** Findings of included MRI studies in humans

Author, Year	Methodology	Groups compared in study	Sample	Age span (years)	Study‐specific exclusion criteria^a^	Results	Risk of bias (volunteer, popularity, or selection bias)	Reference (DOI)
Sugimoto et al., 2018	MRI: 3T Movement artifact elimination: during image processing Blood analysis: ELISA	MDD vs. HC	35 MDD (17 f, 18 m) 35 HC (13 f, 22 m)	20–65 in patients, 20–73 in HC	Treatment with drugs directly effecting the immune system (Steroids, NSAIDs)	FA↓ in IFOF, genu of the CC FA significantly inversely correlated with IL‐1ß levels.	*N* (*n* = 35) Volunteer Popularity Selection	https://doi.org/10.1038/s41398‐018‐0174‐y
Jiang et al., 2018	MRI: 3T Movement artifact elimination: restricting cushions and earplugs Blood analysis: ELISA	MDD vs. HC	98 MDD (39 m, 63 f) 80 HC (38 m and 43 f)	18–45	History of brain injury that led to loss of consciousness	MDD: FA↓ in WM of the bilateral thalamus, right HC, right temporal lobe, left pulvinar Serum MOG and MAG↑ Correlations in MDD patients: MOG and MAG ↔ FA and MD in the WM of the left middle frontal lobe, right inferior frontal lobe, and right SMA	Selection Popularity Volunteer	https://doi.org/10.1016/j.jad.2018.02.044
Yang et al., 2017	MRI: 3T Movement artifact elimination: during image analysis	MDD vs. HC	30 MDD (16 m and 14 f) 28 HC (15 m and 13 f)	18–45	HDRS < 20	FA↓ in left cingulum and FMi Correlation: Mean FA ↔ consummatory anhedonia	*N* (*n* = 35) Volunteer Popularity Selection	https://doi.org/10.1016/j.pscychresns.2017.04.005
Won et al., 2017	MRI: 3T Movement artifact elimination: during image processing Blood analysis: ELISA	MDD vs. HC	42 MDD (11 m and 31 f) 57 HC (20 m and 37 f)	21–65	Comorbid cerebrovascular diseases	MDD: FA↓ in FMa, left ILF AD ↓ in left SLF Correlation:Negative Correlation between SP and FA of FMi of the CC and positive correlation between SP and RD and MD of the right CST	Volunteer Popularity Selection	https://doi.org/10.1038/s41598‐017‐10100‐y
Srivastava et al., 2016	MRI: 3T Movement artifact elimination: during image processing	MDD vs. HC	15 MDD (7 m and 8 fem) 15 HC (6 m and 9 f)	18–45	Family history of psychiatric disorders Mini‐mental State Examination Score lower than 24	FA↓ in left SLF, PFC, parietal region, HC	*N* (*n* = 15) Volunteer Popularity Selection	https://doi.org/10.1176/appi.neuropsych.15050120
Won et al., 2016	MRI: 3T Movement artifact elimination: during image acquisition	MDD vs. HC	35 MDD (10 m and 25 f) 49 HC (15 m and 34 f)	21–64		Body of CC: ‐ FA↓, AD↓ ‐ RD↑ Genu of CC: ‐ FA↓, RD↑ SLC6A4 methylation ↑ MDD Inverse correlation SLC6A4‐methylation↔FA, AD	Volunteer Popularity Selection	https://doi.org/10.1038/tp.2016.137
Cheng et al., 2014	MRI: 1.5T Movement artifact elimination: birdcage head coil and restraining foam pads	EO MDD vs. LO MDD vs. HC	61 MDD (17 m and 44 f) 61 HC (17 m and 44 f)	18–45	HAM‐D score 16 or lower	EO MDD vs. HC: FA↓ in the left ILF A↑ in CC, left IFOF, right FMa, right OR, right corticospinal tract in the midbrain LO MDD vs. HC: FA↓ in IFOF bilaterally, left PLIC, right posterior corona radiata, right ILF, right superior thalamic radiation Correlations between FA and HDRS: EO: Positive correlations: Left corticospinal/corticopontine tract in the midbrain, ILF Negative correlation: HDRS and mean FA in the left ILF and in the SLF bilaterally LO: negative correlations between HDRS and mean FA in FOF, right UF, right anterior corona radiata, left IC, right SLF, left fornix, right ALIC, right cingulum, and right posterior corona radiata	Selection Popularity Volunteer	https://doi.org/10.1371/journal.pone.0112307
Guo et al., 2012	MRI: 1.5T Movement artifact elimination: during image analysis	MDD vs. HC	22 MDD (12 m and 10 f) 19 HC (10 m and 9 f)	18–50	Current illness duration > 6 months; HDRS < 18	FA ↓ in anterior corona radiata, IC, right EC, genu of CC	*N* (*n* = 22) Volunteer Popularity Selection	https://doi.org/10.1016/j.neulet.2012.06.027
Ouyang et al., 2011	MRI: 1.5T Movement artifact elimination: restricting foam pads	MDD vs. HC	18 MDD (9 m and 9 f) 18 HC (9 m and 9 f)	18–45	HDRS < 17	FA↓ in medial frontal gyri bilaterally, temporal lobes, left middle frontal, and cingulate gyri	*N* (*n* = 18) Volunteer Popularity Selection	ISSN: 2078–9947
Wu et al., 2011	MRI: 1.5T Movement artifact elimination: restricting foam pads	MDD vs. HC	23 MDD (10 m and 13 f) 21 HC (9 m and 12 f)	18–45	HDRS under 17 Presence of mood disorders in a first‐degree family member	MDD: FA↓ in right SLF, right frontal lobe, left parietal lobe WM	*N* (*n* = 23) Volunteer Popularity Selection	https://doi.org/10.1016/j.pscychresns.2010.09.002
Zhou et al., 2011	MRI: 3T Movement artifact elimination: birdcage head coil with foam padding	Treatment responsive MDD patients vs. treatment resistant MDD patients	15 Treatment resistant MDD (12 m and 8 f) 20 Treatment responsive MDD (12 m and 8 f)	21–50	HDRS under 18 Cardiovascular disease Acute suicidal or homicidal tendencies	FA↓ bilaterally in treatment resistant patients	*N* (*n* = 15) Volunteer Popularity Selection	https://doi.org/10.1111/add.14596

*Note*: The main methodology and parameters concerning the sample composition and results and risk of bias per study are given. Moreover, study‐specific exclusion criteria in addition to the review's exclusion criteria are listed.

Abbreviations: ALIC, anterior limb of the internal capsule; CC, corpus callosum; EO, early onset; F, female; FMa, forceps major; FMi, forceps minor; FOF, fronto‐occipital fasciculus; HC, healthy controls; HDRS, Hamilton Depression Scale; IC, internal capsule; IFOF, inferior fronto‐occipital fasciculus; ILF, inferior longitudinal fasciculus; ILF, inferior longitudinal fasciculus; LO, late onset; M, male; MDD, major depressive disorder; *N*, small sample size; OR, optical radiation; PFC, prefrontal cortex; PLIC, posterior limb of the internal capsule; SLF, superior longitudinal fasciculus; SMA, supplementary motor area; SP, substance P.

^a^ In addition to the specific exclusion criteria, general exclusion criteria in all studies were as follows: inability to undergo MRI scanning, left‐handedness, presence of a neurological condition, history of other axis I psychiatric or neurological disorders (including substance use disorder), severe somatic diseases.

**TABLE 2:** Findings of included MRI studies in animals

Author, Year	Methods	Groups compared in study	Sample	Results	Risks of Bias	Reference (DOI)
Grandjean et al., 2016	MRI: 9.4T Movement artifact elimination: sedation; image processing	CPS vs. controls	Young adult ♂ C57BL/6 mice (26 CPS; 27 Control)	CPS mice: FA↑ in Cingulum FC↑ amygdala → PFC FC↑ amygdala → cingulate cortex FC↑ in DMN	1 strain 1 age group 1 sex studied (♂)	https://doi.org/10.1016/j.neuroimage.2016.08.013
Zalsman et al., 2017	MRI: 7T Movement artifact elimination: sedation; image processing	WIS+CPS vs. WIS vs. WKY+CPS vs. WKY	Young adult ♂ WIS and WKY rats (20 WIS and 20 WKY)	WKY: FA ↓ in CC, AC MD↑ in CC, FOR	1 age group 1 sex (♂)	https://doi.org/10.1080/15622975.2016.1190866
Coplan et al., 2016	MRI: 3T Movement artifact elimination: anesthesia and Styrofoam headrest	VFD vs. controls	21 young adult ♂ bonnets (12 VFD, 9 control)	Correlation between FA of ALIC and anterior CC in CTRL, no correlation in VFD Correlation between FA of PLIC and posterior CC in VFD, no correlation in CTRL	1 age group 1 sex (♂) *N* (12 macaques)	https://doi.org/10.1016/j.jad.2015.11.049
Kumar et al., 2014	MRI: 7T Movement artifact control: anesthesia for imaging	CMS vs. controls	20 ♂ SD rats (10 CMS, 10 control)	CMS rats: MD↑ in FrCo, CC, left Hippocampus, right cerebral peduncle, left Hypothalamus MD↓ in cingulum FA↓ in FrCo, Hypothalamus, CC Negative correlation between 1st minute OF activity and AD in thalamus. Positive correlation between 1st minute activity and RD in hippocampus	1 strain 1 age group 1 sex (♂) *N* (20 rats)	https://doi.org/10.1016/j.neuroscience.2014.05.037
Van der Marel et al., 2013	MRI: 4.7T Movement artifact control: anesthesia for imaging	5‐HTT KO WIS vs. WT WIS	31 ♂ WIS rats (13 WT, 18 KO)	FA↓ in genu of CC	1 strain (WIS) 1 sex (♂) *N* (18 rats)	https://doi.org/10.1371/journal.pone.0057780
Coplan et al., 2010	MRI: 3T Movement artifact elimination: anesthesia and Styrofoam headrest	VFD vs. controls	21 young adult ♂ bonnets (12 VFD, 9 control)	VFD: FA↓ in ALIC	1 age group 1 sex studied (♂) *N* (12 macaques)	https://doi.org/10.1016/j.neulet.2010.06.012

*Note*: The animals studied and parameters concerning the sample composition and results are given. Moreover, risks of bias are mentioned per study.

Abbreviations: AC, anterior commissure; CMS, chronic mild stress; CPS, chronic psychosocial stress; DMN. default mode network; FA, fractional anisotropy; FC, functional connectivity; FOR, fornix; FrCo, frontal cortex; MD, mean diffusivity; *N*, small sample size; SD, Sprague–Dawley; VFD, variable foraging demand; WIS, Wistar rats; WKY, Wistar–Kyoto rats.

### 3.1 Interhemispheric connectivity

One of the regions displaying replicable significant FA reductions in MDD is the corpus callosum. This structure contains commissural fibers, transmitting information between the two hemispheres. The most common classification of the corpus callosum, first proposed by Witelson, subdivides the corpus callosum into seven subsections, referred to as CC1–CC7 (Witelson, 1989). As different parts of the corpus callosum connect different brain regions, this categorization allows for a correlation of subsections with brain functions (Hofer & Frahm, 2006; Witelson, 1989). Structures of the prefrontal and frontal lobes are connected by the rostrum (CC1), the genu (CC2), and the rostral part of the body of the corpus callosum (CC3) (Hofer & Frahm, 2006). The anterior and posterior midbodies (CC4 and CC5) are associated with sensorimotor connections, the isthmus (CC6) is associated with mid‐temporal connections, and the splenium (CC7) with occipital regions (Hofer & Frahm, 2006). Besides the structures of the corpus callosum, the forceps minor connecting the frontal lobes, as well as the forceps major connecting the occipital lobes, can be delineated as further structures establishing interhemispheric connectivity (Trepel & Dalkowski, 2017). Reviewed studies reported a significantly reduced FA in the genu (CC2) (Guo et al., 2012; Sugimoto et al., 2018; Won et al., 2016) and the body of the corpus callosum (CC3–5) (Won et al., 2016) as well as in the forceps minor (Yang et al., 2017), suggesting mainly impaired interhemispheric connectivity between frontal lobes in medication‐naïve MDD patients. Moreover, a significantly decreased FA in the forceps major of untreated MDD patients could be identified, implying impaired occipital connectivity (Won et al., 2017). Findings of impaired interhemispheric connectivity are further supported by alterations concerning other diffusion tensor imaging parameters, as a decreased FA was accompanied by an increased radial diffusivity as well as a decreased axial diffusivity in treatment‐naïve MDD patients, both findings indicating impaired integrity of the corpus callosum (Won et al., 2016). Furthermore, Cheng et al. could show that early‐onset MDD patients (defined as having the first depressive episode before the age of 30) had an increased FA in the corpus callosum as well as in the right forceps major compared to age‐matched healthy controls (Cheng et al., 2014). Interestingly, late‐onset MDD patients (defined as having the first depressive episode above the age of 30) showed no differences in the FA of the corpus callosum or the forceps major or forceps minor compared to age‐matched controls (Cheng et al., 2014).

Animal studies could confirm that alterations concerning the integrity of the corpus callosum in MDD are present across different species. Zalsman et al. investigated whether Wistar–Kyoto rats, a depressive and anxious‐like breed, show WM alterations compared to control Wistar rats. In Wistar–Kyoto rats, a decreased FA and an increased mean diffusivity in the corpus callosum as well as decreased FA in the left and right anterior commissures compared to Wistar rats were found, indicating impaired interhemispheric frontal connectivity (Zalsman et al., 2017). Another study investigating the effects of genetic alterations on WM integrity has been carried out by Van der Marel et al., where the influence of a knockout of the serotonin transporter gene *SLC6A4* was investigated in rats. This study identified a significantly reduced FA in the genu of the corpus callosum of knockout rats (van der Marel et al., 2013). Reduced expression of this gene has been previously linked to depressive disorders both in rodents (Olivier et al., 2008) and in humans (Bleys et al., 2018). Moreover, in MDD patients, a significantly increased *SLC6A4* methylation was found, along with a significant inverse correlation between *SLC6A4* DNA methylation and FA, as well as with axial diffusivity of the corpus callosum (Won et al., 2016). These findings imply a structural effect of *SLC6A4* expression on the integrity of the corpus callosum, supporting the hypothesis that alterations in serotonin homeostasis could precede depressive behavior and impaired connectivity.

Besides genetic models of depression, chronic mild stress exposure has been shown to cause impaired interhemispheric connectivity in rodents as well. In this context, Kumar et al. (2014) demonstrated a significant decrease of FA in the corpus callosum of Sprague–Dawley rats following chronic stress. Moreover, animals subjected to chronic stress also exhibited less weight gain and had a lower sucrose intake in the sucrose preference test, as well as increased immobility in the forced swim test, indicating that the detected impaired interhemispheric connectivity was paralleled by a depressive‐like phenotype (Kumar et al., 2014). Furthermore, not only human and rodent studies but also studies conducted on primates have revealed results implicating impaired interhemispheric connectivity in MDD. In a study by Coplan et al., depression was induced in macaques using a Variable Foraging Demand protocol, whereby mothers were forced to spend more time away from their offspring, thus inducing stress for the youngsters. This study found that while there was concordance between the FA of the anterior corpus callosum and the anterior limb of the internal capsule in macaques growing up under normal conditions, these values were discordant in the experimental group (Coplan et al., 2016). In contrast to that, an FA concordance between the posterior limb of the internal capsule and the posterior corpus callosum as well as between occipital WM and the posterior corpus callosum could be found in the experimental group, but not in the control group (Coplan et al., 2016). These findings imply impaired WM integrity in frontal interhemispheric connectivity following early life stress and might indicate a disruption in the synchronous development of myelination in frontal WM (Coplan et al., 2016).

In summary, reviewed studies highlight the crucial role of intact interhemispheric connectivity, pointing to the fact that impairments, especially in FA, are consistently found in patients suffering from MDD as well as in animal models of depression.

### 3.2 Association tracts

In addition to assessing commissural fibers, several studies have investigated whether altered diffusion metrics in association tracts can be detected in patients with MDD, as well as in animal models of depression.

#### 3.2.1 Cingulum bundle

The cingulum bundle is a highly complex WM tract, connecting the anterior thalamic nuclei, the cingulate gyrus, and the parahippocampal region, thus being a crucial component of the Papez circuit (Bubb et al., 2018). Short and long association fibers, as well as fibers radiating across the cingulum bundle, aiming to reach numerous cortical and subcortical structures, make the cingulum bundle an exceptionally versatile structure (Bubb et al., 2018). Most fiber tracts enter the cingulum only to exit it shortly afterward, with only a few fiber tracts running the entire extent of the cingulum bundle (Heilbronner & Haber, 2014). This makes the cingulum bundle highly sophisticated and diverse, containing fiber tracts associated with different functional entities. While the functions of the cingulum bundle are manifold, there is consensus that its main functions comprise executive control, emotion, pain, and episodic memory, as recently reviewed by Bubb et al. (2018).

In studies included in the current review, unmedicated patients with MDD expressed a significantly decreased FA in the left cingulum (Yang et al., 2017). No other significant alterations in WM metrics of unmedicated MDD patients were found. In rodents, the left and right cingulum of Wistar–Kyoto rats have both shown a nonsignificant trend toward decreased FA compared to Wistar rats (Zalsman et al., 2017). Contradictorily, results indicating increased connectivity have also been reported. Rats subjected to chronic mild stress showed a trend towards a nonsignificant decrease of mean diffusivity in the bilateral cingulum (Kumar et al., 2014). In mice subjected to chronic psychosocial stress, a significant increase in the FA of the cingulum could be detected (Grandjean et al., 2016).

The fact that findings concerning the cingulum bundle show conflicting results might be attributed to the versatility of this tract, as well as to the still unclarified role of different segments of this bundle. Moreover, it is important to note that the cingulum bundle contains numerous crossing fibers, thus possibly interfering with measurements of diffusion metrics (Bubb et al., 2018).

#### 3.2.2 Longitudinal and fronto‐occipital fasciculi

The superior longitudinal fasciculus connects frontal cortical regions with posterior parietal cortical areas, thus playing an important role in spatial attention and integration of environmental influences as well as responsive motor behavior (Petrides & Pandya, 2012; Vecera & Rizzo, 2003). Reduced connectivity, as detected by reduced FA in diffusion tensor imaging, might thus play a role in the development of psychomotor retardation as one of the main symptoms of MDD (Tolentino & Schmidt, 2018). Fibers contained in the inferior longitudinal fasciculus and the inferior fronto‐occipital fasciculus terminate at neurons of the occipital lobe, the inferior longitudinal fasciculus connecting this region with the temporal lobe, and the inferior fronto‐occipital fasciculus connecting it with the frontal cortex, whereby these tracts show a relevant spatial overlap along a major part of their pathway (Ashtari, 2012). Concerning the role of these tracts in MDD, a lower FA in the inferior longitudinal fasciculus is correlated with impaired cognitive flexibility, a common symptom in depression (Chanraud et al., 2010).

In treatment‐naïve MDD patients, a decreased FA was found in the left superior longitudinal fasciculus (Srivastava et al., 2016; Zeng et al., 2021) as well as in the right superior longitudinal fasciculus (Wu et al., 2011; Zeng et al., 2021). Also, a decreased FA could be identified in the inferior fronto‐occipital fasciculus (Sugimoto et al., 2018) and the left inferior longitudinal fasciculus (Won et al., 2017). Moreover, age‐related alterations in these tracts could be determined, as early‐onset MDD patients exhibited a decreased FA in the left inferior longitudinal fasciculus and an increased FA in the left fronto‐occipital fasciculus (Cheng et al., 2014). Interestingly, a decreased FA in the left inferior longitudinal fasciculus could no longer be detected when early‐onset patients with an onset age of 26–29 years were excluded from the calculations. Late‐onset patients, on the other hand, expressed a decreased FA in the inferior fronto‐occipital fasciculus bilaterally, as well as in the right inferior longitudinal fasciculus (Cheng et al., 2014). In this study, excluding patients close to the delineation of early‐ and late‐onset depression resulted in increased FA in association tracts showing alterations in early‐onset MDD and decreased FA in late‐onset MDD. Excluding patients close to the delineation makes the differentiation between the two subgroups clearer and could thus point to different etiologies and pathophysiologies acting in the development of early‐ and late‐onset MDD (Cheng et al., 2014).

Concerning animal models of depression, Grandjean et al. (2016) induced chronic psychosocial stress in mice and detected aberrant diffusion metrics in WM. To specify stress‐induced alterations, measurements from before and after the stress paradigm were compared. Stress increased functional connectivity in the default mode network in prefrontal and cingulate cortices, as well as in the amygdala–cingulate cortex network (Grandjean et al., 2016).

The relevance of alterations in association tracts is highlighted by translational findings in rodents and humans. Future studies investigating association tracts should focus on the role of the inferior longitudinal fasciculus and the inferior fronto‐occipital fasciculus in the development of psychomotor retardation. In animal models of depression, this could, for example, be realized by correlating FA in these tracts with activity in the open‐field test, a common test used to measure motor activity and exploratory behavior in rodents (Gould et al., 2009). In humans, diffusion tensor imaging metrics should be correlated with reaction time measurements, a test generally considered to assess psychomotor activity (Buyukdura et al., 2011; Hickie et al., 1999).

### 3.3 Projection tracts

In humans, fibers from different frontal‐subcortical circuits converge into the internal capsule (Guo et al., 2012). Among these circuits are the orbitofrontal circuit, the dorsolateral prefrontal circuit, as well as the anterior cingulate circuit, which have been associated with impaired emotional stability, executive function, and motivation, respectively, and have thus been associated with the pathogenesis of MDD (Guo et al., 2012; Rogers et al., 1998; Zhu et al., 2011). While most of the internal capsule contains afferent and efferent fibers connecting the cortex and the spinal cord, the anterior limb of the internal capsule mostly contains fibers that reciprocally connect the thalamus and the frontal lobes. A bilateral surgical interruption of the thalamocortical radiation arising from the anterior limb of the internal capsule has been shown to result in personality changes resembling the symptoms of MDD, even without damage to the cortex (Freeman & Watts, 1942).

Several alterations in projection tracts linking cortical and subcortical structures have been reported. In treatment‐naïve MDD patients, decreased FA has been found in the anterior corona radiata (Guo et al., 2012), the internal capsule (Cheng et al., 2014; Guo et al., 2012) as well as the right external capsule (Guo et al., 2012), the right superior thalamic radiation (Cheng et al., 2014), and in the right posterior corona radiate (Cheng et al., 2014). Interestingly, an increased FA has been found in early‐onset patients in the optical radiation as well as in the right corticospinal tract (Cheng et al., 2014). In addition, one study identified a significant negative correlation in late‐onset MDD patients between points reached on the Hamilton Rating Scale for Depression and FA in the right anterior and the right posterior corona radiata, the left external capsule, and the right anterior limb of the internal capsule indicating that more severe depression is associated with a more pronounced impairment of WM in these regions (Cheng et al., 2014). The same study found a positive correlation between FA in the left corticospinal and corticopontine tracts in the mesencephalon and Hamilton Rating Scale score in early‐onset MDD patients, possibly indicating more pronounced connectivity in these tracts in more severe depression (Cheng et al., 2014).

In rodents, increased mean diffusivity, indicating reduced connectivity, has been found in the right cerebral peduncle in rats following stress (Kumar et al., 2014). Moreover, reduced FA in the anterior limb of the internal capsule but no changes in the posterior limb have been reported in male bonnet macaques exposed to early life stress (Coplan et al., 2010).

Reproducible alterations in frontal‐subcortical circuits thus support the hypothesis that damage to the WM of the internal capsule plays an important role in the development of mood disorders. Based on these translational findings, further research is required to determine how errant myelination of the anterior limb of the internal capsule after early life stress in humans is involved in the emergence of MDD.

### 3.4 Gyral WM

While gyral WM fulfills important functions in physiological brain activity, several characteristics of this area render it difficult to be examined using diffusion tensor imaging. It is important to note that gyral WM contains numerous U‐shaped fibers, as well as pyramid‐shaped crossings, which can interfere with water diffusion, thus mimicking a higher fiber density in diffusion tensor imaging metrics (Oouchi et al., 2007; Shinohara et al., 2020). Nevertheless, phenomena of crossing, kissing, and recurring fibers are present in patients as well as healthy controls and thus, reproducible findings of decreased FA in gyral WM are unlikely to solely originate from artifacts.

In treatment‐naïve MDD patients, FA reductions in gyral WM of the left prefrontal cortex (Srivastava et al., 2016), the left parietal region (Srivastava et al., 2016; Wu et al., 2011), medial frontal gyri (Ouyang et al., 2011), the right temporal lobe (Ouyang et al., 2011), the left middle frontal gyrus, as well as of cingulate gyrus have been identified (Ouyang et al., 2011). Moreover, Jiang et al. revealed correlations between levels of Myelin Oligodendrocyte Glycoprotein (MOG) as well as Myelin‐Associated Glycoprotein (MAG) in serum, as well as FA and mean diffusivity in the WM of the frontal lobe bilaterally in treatment‐naïve MDD patients, but not in healthy subjects (Jiang et al., 2018). MOG and MAG levels in serum were also found to be significantly elevated in MDD patients compared to healthy controls (Jiang et al., 2018). Although the proteins MOG and MAG are relatively minor components of the myelin sheath, they have been suggested to play an important role in demyelination (Jiang et al., 2018). MAG release, for example, is particularly pronounced in early myelination (Jiang et al., 2018). Moreover, demyelination in the context of autoimmune diseases due to antibodies produced against MOG and MAG has been identified (Amor et al., 1994). These findings show that even though demyelinating diseases, such as multiple sclerosis, are characterized by different symptoms than depression, demyelination as a possible process contributing to the pathogenesis of MDD should not be discarded.

## 4 POSTMORTEM FINDINGS CONCERNING WM ALTERATIONS

In the second part of this review, the cellular and molecular correlates of WM alterations will be investigated. For an overview of all included postmortem studies discussed in this section, see Tables 3 and 4. Postmortem studies of WM alterations can be a valuable resource to identify the underlying pathomechanism of MDD. Of note, due to the high prevalence of comorbid substance use disorder in victims of suicide, it was not possible to exclude this factor as a possible confounder.

**TABLE 3:** Findings of included postmortem studies in humans

Author, Year	Methodology	Groups compared in study	Sample	Study specific exclusion criteria^a^	Results	Risks of bias (volunteer, popularity, or selection bias)	Reference (DOI)
Tanti et al., 2018	Investigation of OL in ventromedial prefrontal WM in postmortem brain tissue	MDD + CA vs. MDD w/o CA vs. controls	18 MDD patients with CA (all m); age 26–48 years, 18 DoS 18 MDD patients w/o CA (all m); 32–54 years, 18 DoS 18 controls (all m); age 23–52 years. No CA, no DoS	Cause of death other than suicide in MDD	CA is associated with an increased number of mature OL CA is associated with a decreased number of immature OL Increased expression of the TF MASH1 Effects were absent in MDD patients without CA	Selection *N* (18 patients per group)	https://doi.org/10.1038/mp.2017.231
Hamazaki et al., 2017	Investigation of the CC in postmortem brain tissue	MDD vs. controls	15 MDD patients (8 m and 7 f); age range 45–69, 13 DoS 15 Controls (8 m and 7 f); age range 44–70, no DoS		No significant differences in the levels of PUFA or other fatty acids between controls and MDD	Selection *N* (15 patients per group)	https://doi.org/10.1016/j.eurpsy.2016.05.007
Lutz et al., 2017	Investigation of postmortem brain samples of humans	MDD + CA vs. MDD w/o CA vs. controls	26 controls (20 m and 6 f); age range 15–81 years, no DoS 27 MDD patients with CA (20 m and 6 f); age range 19–85 years, 27 DoS 25 MDD Patients w/o CA (22 m and 3 f); age range 18–77 years, 25 DoS	MDD Patients who have died of a cause other than suicide	OL density ↓ in WM of cingulate cortex in MDD+CA, not in MDD w/o CA Axonal diameter↓, myelin thickness↓, g‐ratio ↑ in MDD+CA Methylation of LINGO3, POU3F1 ↓ in MDD+CA, not in MDD w/o CA ITGB1 mRNA ↓ in MDD+CA Strong correlation between myelin gene expression changes Correlation between myelin gene expression in LLG rats and CA humans	Selection	https://doi.org/10.1176/appi.ajp.2017.16111286
Szebeni et al., 2017	Investigation of postmortem brain tissue	MDD vs. controls	18 MDD patients (all m); age range 16–86 years, 14 DoS 18 controls (all m); age range 17–82, none DoS		DNA oxidation ↑ in WM of BA10 in MDD patients DNA repair enzymes PARP1 and OGG1 ↑ in OL of MDD patients DNA oxidation↑ in WM of anhedonic rats	Selection	https://doi.org/10.1093/ijnp/pyw114
Miguel‐Hidalgo et al., 2017	Investigation of postmortem brain tissue in humans and rats	Controls vs. MDD	Humans: 10 MDD patients (5 m and 5 f); age range 43–49 years, 8 DoS 10 Controls (8 m and 2 f); mean age 47–55 years, none DoS		MDD patients: miR21 ↓ in prefrontal WM miR21 expression positively correlated with expression of myelin‐related mRNA miR21KO Mice: MBP intensity in ACC ↓ Premyelinating OL in CC ↑	Selection *N* (10 patients)	https://doi.org/10.1016/j.pnpbp.2017.08.009
Rajkowska et al., 2015	Investigation of the WM of the PFC in postmortem brain tissue	MDD vs. controls	20 MDD patients (11 m and 9 fem); age range 20–87, 15 DoS 16 controls (10 m and 6 f); age range 27–80, none DoS	Evidence of head trauma Neurological diseases Cause of death other than suicide in MDD	OL soma size in gyral WM of MDD patients significantly ↓ Brain tissue from control rhesus monkeys suggests no direct effect of antidepressant medication on OL morphology *PLP1*‐mRNA‐expression ↓ in MDD mRNA‐Expression of *CNP*, *MOG*, *Olig1* ↑ in MDD	Selection *N* (20 patients)	https://doi.org/10.1016/j.jpsychires.2015.04.010
Torres‐Platas et al., 2011	Investigation of astrocytes in postmortem brain tissue	MDD vs. controls	10 MDD patients (7 m and 3 f); age range 43–53 years, 10 DoS 10 controls (8 m and 2 f); age range 42–55, no DoS	Cause of death other than suicide in MDD	Cell size, cell length, process density, and length ↑ in MDD	Selection *N* (10 patients)	https://doi.org/10.1038/npp.2011.154

*Note*: The main methodology and parameters concerning the sample composition and results and risk of bias per study are given. Moreover, study specific exclusion criteria in addition to the review's exclusion criteria are listed.

^a^ In addition to the specific exclusion criteria, general exclusion criteria in all studies were as follows: presence of other neurological other psychiatric disease, no consent given by closest living relative.

Abbreviations: CA, childhood abuse; CC, corpus callosum; DoS, died of suicide; LG, licking and grooming; MDD, major depressive disorder; *N*, small sample size; OL, oligodendrocytes; PUFA, polyunsaturated fatty acids; WM, white matter; w/o, without.

**TABLE 4:** Findings of included postmortem studies in animals

Author, Year	Groups compared in study	Sample	Results	Risks of bias	Reference (DOI)
Gao et al., 2019	CUS+FLUOX vs. CUS w/o FLUOX vs. control	Adult male SD rats (10–12 weeks old)	CUS w/o FLUOX vs. control Total length of myelinated fibers ↓ Total volume of myelinated fibers ↓ Mean volume of WM ↓	1 strain 1 sex (♂) 1 age group	https://doi.org/10.1016/j.neulet.2018.11.013
Cathomas et al., 2019	CSS vs. control *CNP1* ^+/−^ + CSS vs. *CNP* wild‐type CSS	Adult male C57BL/6 mice (12–13 weeks old)	CSS vs. control OL‐related gene expression in PFC and AMY WM ↓ *CNP1* ^+/−^ + CSS vs. *CNP* wild‐type CSS Social interaction ↓ Microglia activity ↑	1 strain Small sample size (*N* = 12)	https://doi.org/10.1111/gbb.12475
Xiao et al., 2018	DEP + exercise vs. DEP w/o exercise vs. control	Adult male SD rats	DEP w/o exercise vs. control Sucrose preference ↓ Length and volume of myelinated fibers ↓ Thickness of myelin sheath ↓ DEP+ exercise vs. DEP w/o exercise Sucrose preference ↑ Length and volume of myelinated fibers ↑ Thickness of myelin sheath ↑	1 strain 1 sex (♂)	https://doi.org/10.1002/cne.24350
Gao et al., 2017	CUS vs. control	Adult male SD rats (10–12 weeks old)	In CUS: MBP ↓ Total WM volume ↓ Total fiber length ↓ Mean diameter ↓	1 strain 1 sex (♂) 1 age group	https://doi.org/10.1002/cne.24178
Chen et al., 2016	CUS + exercise vs. CUS w/o exercise vs. control	Adult male SD rats	CUS w/o exercise vs. control Total WM volume ↓ Total WM capillary volume ↓ Total WM capillary length ↓ Total WM capillary surface area ↓ No significant difference between CUS + exercise vs. control	1 strain 1 sex (♂)	https://doi.org/10.1002/cne.24017
Miyata et al., 2016	CUS vs. control	Adult male C57/BL6 mice (11 weeks old)	In CUS: NoR and PoR narrower in the CC CASPR expression ↓ Neurofascin ↓	1 strain 1 sex (♂) 1 age group	https://doi.org/10.1038/srep23084
Wang et al., 2014	CMS+DVFX vs. CMS w/o DVFX vs. control	Adult female C57/BL6 mice (6 weeks old)	CMS w/o DVFX vs. control Myelin‐related proteins ↓ OL‐related proteins ↓ Sucrose consumption ↓ Mobility time in TST ↓ Mobility time in FST ↓ No significant difference between CMS+ DVFX vs. control	1 strain 1 sex (♀) 1 age group	https://doi.org/10.1111/jnc.12792
Hagemeyer et al., 2012, ^a^	*CNP* ^+/−^ vs. wild‐type	Adult mice (4–26 months old)	*CNP* ^+/‐^ mice: Astrocytes in CC ↑ Microglia in CC ↑ T‐Lymphocytes in CC ↑ APP ↑ All alterations age dependent	1 strain	https://doi.org/10.1002/emmm.201200230
Gosselin et al., 2009	WKY rats vs. SD rats	Adult male SD and WK rats	No significant difference in the number of astrocytes between WK and SD rats	1 rat strain as control (SD) 1 sex (♂)	https://doi.org/10.1016/j.neuroscience.2008.10.018

*Note*: The animals studied and parameters concerning the sample composition and results are given. Moreover, risks of bias are listed per study.

^a^ The publication includes a part that has looked at humans. This part was not included in our review since it interferes with our exclusion criteria. The mouse studies were conducted independently of the human studies and are eligible.

Abbreviations: CC, corpus callosum; CSS, chronic social stress; CUS, chronic unpredictable stress; DEP, depressive phenotype; DVFX, desvenlafaxine; FLUOX, fluoxetine; NoR, nodes of Ranvier; w/o, without; PoR, paranodes of Ranvier; SD, Sprague–Dawley; WKY, Wistar–Kyoto.

### 4.1 Oligodendrocyte density and morphology

Several studies have investigated alterations concerning the density and morphology of oligodendrocytes in gyral WM. A reproducible finding in this context is a reduced density of oligodendrocytes in gyral WM of MDD patients following childhood abuse, defined as severe sexual or physical abuse before the age of 15 (Lutz et al., 2017; Tanti et al., 2018). Childhood abuse is considered an important risk factor for the development of MDD, as reviewed by Carr et al. (2013). In addition to findings of reduced oligodendrocyte density, a recent study by Tanti et al. yielded more profound results concerning the influence of childhood abuse on different aspects of oligodendrocyte integrity. Accordingly, three groups of patients were defined: MDD patients with a history of childhood abuse; those with MDD, but without having experienced childhood abuse; and control patients, who died of a different reason than suicide and have not had any neurological or psychiatric illnesses during their lifetime. Oligodendrocytes were identified using immunohistochemistry staining against Olig2, a protein expressed throughout all stages of development and exclusively by this cell type (Tanti et al., 2018). MDD patients with a history of abuse presented a significantly lower density of oligodendrocytes compared to MDD patients without childhood abuse, as well as to healthy controls (Tanti et al., 2018). Interestingly, no significant difference between MDD patients and healthy controls was found, implying that not depression itself but childhood abuse was the driving factor behind altered Olig2+ cell density (Tanti et al., 2018). This confirms findings first reported by Lutz et al., who identified a significant decrease in total oligodendrocyte density in suicide patients who experienced childhood abuse, but not in those who suffered from MDD without childhood abuse history (Lutz et al., 2017). Moreover, both studies conclude that no significant difference in the number of oligodendrocyte progenitor cells could be detected between the groups as identified by the density of cells expressing platelet‐derived growth factor receptor α (PDGFRα) (Lutz et al., 2017; Tanti et al., 2018). Therefore, it has been suggested that the decrease in Olig2+ cells was unrelated to the pool of immature cells (Tanti et al., 2018). Surprisingly, Tanti et al. found a significant increase in the density of mature oligodendrocytes (identified as Nogo‐A+ cells) in MDD patients with childhood abuse, compared to both patients with MDD but without abuse and healthy controls. This result could be confirmed by measuring the density of APC+ cells, another specific marker for mature oligodendrocytes, showing a strong colocalization with Nogo‐A (Tanti et al., 2018). Since the density of mature oligodendrocytes was significantly increased in the childhood abuse MDD group, while the density of oligodendrocyte progenitor cells showed no significant group differences, the authors hypothesize that the significant reduction in the total number of oligodendrocytes following childhood abuse is caused by a reduction in the number of cells not expressing mature oligodendrocyte markers yet, but also not expressing oligodendrocyte progenitor cells markers anymore (Tanti et al., 2018). Moreover, an age‐related effect of Olig2 expression in MDD patients with a history of childhood abuse could be identified. A significant correlation between age at the time of death and expression of Olig2 was found, meaning that older patients showed a higher density of oligodendrocytes than younger patients (Tanti et al., 2018). On the other hand, a significant negative correlation between the number of Nogo‐A+ cells and age was found, implying that the number of mature oligodendrocytes decreased with a higher age at suicide (Tanti et al., 2018). It can therefore be hypothesized that a recovery of the Olig2+ cell population might take place with progressing age (Tanti et al., 2018). To further investigate the maturation of oligodendrocytes, the authors utilized staining against SOX10. This protein is expressed continuously in oligodendrocytes, though stronger in immature ones than in mature myelinating oligodendrocytes (Tanti et al., 2018). The density of Nogo‐A‐positive oligodendrocytes showing high SOX10 expression, deemed an intermediate phenotype, was significantly lower in MDD patients with childhood abuse than in controls (Tanti et al., 2018). Taken together, these findings suggest a more mature phenotype of oligodendrocytes in patients having experienced childhood abuse. To closer examine oligodendrocyte differentiation, staining against mammalian achaete‐scute homolog‐1 (MASH1), a protein that has been shown to play a critical role in the process of oligodendrocyte progenitor cells differentiation and maturation (Nakatani et al., 2013; Parras et al., 2007), was used. In the group with MDD patients having a history of childhood abuse, a significant increase in MASH1 expression could be detected, compared to MDD patients without childhood abuse and controls. However, no significant difference between MDD patients and controls could be identified (Tanti et al., 2018). Moreover, MASH1 expression showed a significant negative correlation with age at the time of death, indicating an increased maturation of oligodendrocytes at the age closest to childhood abuse (Tanti et al., 2018). This finding implies altered myelination profiles in WM, which are specific to childhood abuse, but not to MDD. The authors, therefore, hypothesize that childhood abuse may trigger a maladaptive increase in the rate of differentiating oligodendrocytes (Tanti et al., 2018).

Similarly, Rajkowska et al. investigated WM alterations in postmortem brain samples from both suicidal and nonsuicidal MDD patients and controls. A positive correlation between oligodendrocyte density and age in the MDD group, although not in controls, was found (Rajkowska et al., 2015). Moreover, a significant group difference in the soma size of oligodendrocytes could be identified, with the MDD group expressing approximately 13% smaller values (Rajkowska et al., 2015). However, no group differences concerning oligodendrocyte density and no further correlations could be found (Rajkowska et al., 2015). No significant differences between cell density and cell size of oligodendrocytes—identified by 2ʹ,3ʹ‐cyclic nucleotide 3ʹ‐phosphodiesterase (CNP) immunoreactivity—could be found between MDD and controls. Furthermore, no significant differences between oligodendrocyte density and age, nor oligodendrocyte density and illness duration could be found (Rajkowska et al., 2015).

From a translational perspective, aberrations in oligodendrocyte maturation represent findings across humans and mice. Miyata et al. focused on differentiating between findings concerning mature oligodendrocytes and oligodendrocyte progenitor cells, utilizing immunohistochemistry staining against APC and NG2, respectively. Sholl analysis could identify that processes of mature oligodendrocytes were longer, thicker, and had a higher density in mice having experienced chronic stress, than in controls (Miyata et al., 2016). Chronic stress did not, however, affect the density or the morphology of oligodendrocyte progenitor cells (Miyata et al., 2016). It also did not lead to microglia activation, nor did it increase the number of astrocytes in the corpus callosum (Miyata et al., 2016). Concerning different subtypes of oligodendrocytes, the authors therefore conclude that chronic stress has a stronger effect on mature oligodendrocytes than on oligodendrocyte progenitor cells (Miyata et al., 2016).

The underlying mechanisms responsible for the aforementioned contrasting findings could be attributed to the fact that unlike the studies of Tanti et al. (2018) and Lutz et al. (2017), Rajkowska et al. (2015) have not differentiated between depressed patients who experienced childhood abuse and those who did not. Based on the studies conducted by Tanti et al. and Lutz et al., it can be assumed that alterations in oligodendrocyte density are mainly driven by childhood abuse and not MDD. While studies conducted on rodents focusing on oligodendrocytes in depression are rare, published findings imply that aberrations in oligodendrocyte morphology following chronic stress are comparable to those present in patients having experienced childhood abuse, thus further highlighting the relevance of alterations in oligodendrocyte morphology following adversities.

### 4.2 Findings on a molecular level

Postmortem studies allow for a thorough investigation not only of alterations on a cellular level but also on a molecular level. In this context, studies have investigated alterations in protein expression, mRNA expression, DNA damage, and cell metabolism, both in humans and in rodents.

One aspect that has been investigated is the metabolism of polyunsaturated fatty acids (PUFA) in deep WM. These lipids, along with glycerolipids, glycerophospholipids, and sphingolipids, play a crucial role in forming cell membranes (Müller et al., 2015). Alterations in PUFA metabolism are associated with MDD (Hamazaki et al., 2017; Müller et al., 2015). Specifically, a diet lacking *n*‐3 PUFA has been shown to induce a depressive phenotype in rodents (Müller et al., 2015). In humans, Hamazaki et al. investigated alterations of relative levels of PUFA in the corpus callosum of patients having suffered from MDD using thin‐layer and gas chromatography. No significant difference in relative PUFA levels between MDD patients and healthy controls could be identified.

In the study conducted by Lutz et al. mentioned above, the childhood abuse group expressed a decreased methylation of the *LINGO3* as well as the *POU3F1* gene in oligodendrocytes, but not in neurons (Lutz et al., 2017). The LINGO3 protein belongs to the LINGO family, a group of proteins that have been linked to myelination (Mi et al., 2005), while POU3F1 is a transcription factor controlling myelination (Ryu et al., 2007). Interestingly, while POU3F1 has been shown to promote myelination, LINGO1 seems to negatively influence this process (Mi et al., 2005; Ryu et al., 2007). These results provide evidence for oligodendrocyte‐specific epigenetic alterations as a consequence of childhood abuse, thus offering a possible pathomechanism leading to an altered oligodendrocyte maturation pattern. Transcriptomic differences between the groups were also investigated. A total of 32 genes that have been linked to myelination were downregulated in the childhood abuse group, while three genes were upregulated (Lutz et al., 2017). Downregulated genes coded for essential building blocks of myelin or were genes that control the synthesis of myelin lipids or were responsible for the differentiation of oligodendrocytes (Lutz et al., 2017). This downregulation was only present in the WM of the anterior cingulate cortex, but not in the amygdala, thus suggesting a region‐specific impairment of myelination with a focus on frontal areas (Lutz et al., 2017). This is in line with alterations in the methylation pattern of the anterior limb of the internal capsule, but not in the posterior limb of the internal capsule found in macaques after being exposed to early life stress (Coplan et al., 2016). Despite showing no difference in methylation (Lutz et al., 2017), *ITGB1* mRNA was found to be strongly downregulated in the childhood abuse group. Interestingly, the expressions of *LINGO3* and *POU3F1* mRNA were not decreased, despite these genes being hypermethylated (Lutz et al., 2017). The ITGB1 protein promotes myelination by forming complexes with other integrins (ITGA6 and ITGAV), which were also found to be downregulated in the childhood abuse group (Lutz et al., 2017). Integrins are crucial for adhesion between cells and the extracellular matrix, therefore suggesting that the downregulation of *ITGB1* mRNA is paralleled by an impaired embedding of oligodendrocytes in the surrounding tissue (Lutz et al., 2017). As the downregulation of *ITGB1* mRNA was only present in MDD patients who have experienced childhood abuse, these results may suggest that early life adversities are associated with impaired transcription of this essential myelin gene. This is in line with further studies finding that not MDD but childhood abuse is closely related to alterations in characteristics of oligodendrocytes. Therefore, future research should consider the role of altered *ITGB1* expression in patients who experienced childhood abuse to clarify its role in altered myelination.

Concerning translational findings, Lutz et al. also evaluated behavior and gene expression in the offspring of rat dams displaying high or low levels of maternal care, respectively. A strong correlation between myelin gene expression changes in rats raised by low maternal behavior dams and expression changes in humans who experienced childhood abuse could be determined (Lutz et al., 2017). The authors also utilized spectroscopic methods to further investigate myelination structure. A moderate but significant decrease in axonal diameter in MDD patients with childhood abuse compared to both MDD patients without abuse and the control group could be identified, along with a decrease in myelin thickness in the MDD and childhood abuse, but not in the MDD without abuse group. The g‐ratio (coefficient of axonal thickness to total fiber thickness) was increased in patients with childhood abuse, meaning that the decrease in myelin thickness outweighed the decrease of axonal diameter (Lutz et al., 2017), pointing to demyelination being more pronounced than axonal loss. The results suggest that a low level of maternal care in rodents is a suitable translational model to investigate alterations in the expression of myelin‐related genes in humans having experienced childhood abuse (Lutz et al., 2017). These findings also confirm that childhood adversities may interfere with normal myelination processes across different species.

Findings concerning transcriptomic alterations could be confirmed in other studies. Rajkowska et al. reported a significant decrease in Proteolipid protein 1 (*PLP1*) mRNA in MDD patients, compared to controls. PLP1 is a transmembrane domain protein, which binds copies of itself, thus playing an important role during the wrapping of the myelin sheath. PLP1 defects have been associated with the degeneration of cortical axons in both humans and mice (Garbern et al., 2002). Also, a significant positive correlation between *PLP1* gene expression and oligodendrocyte soma size could be identified, which is in line with other findings reporting a significantly smaller soma size in MDD, without a difference in oligodendrocyte density (Rajkowska et al., 2015). Moreover, the authors speculate that the underlying reason for decreased *PLP1* mRNA expression might be due to PLP1 protein downregulating mRNA synthesis (Rajkowska et al., 2015). On the other hand, a significant upregulation of the mRNA expression of the oligodendrocyte‐enriched genes *CNP*, *MOG*, and *Olig1* could be identified in MDD patients. Concerning proteins, CNP protein expression from subjects with MDD was significantly lower than that in controls, despite showing a significantly higher mRNA expression (Rajkowska et al., 2015). This suggests that it is not only mRNA expression eliciting control over the amount of CNP protein synthesized in oligodendrocytes. Overexpression of *CNP* mRNA has been shown to induce aberrant myelination, leading to accelerated expression of Myelin Basic Protein (*MBP*) and *PLP1* (Gravel et al., 1996), indicating that this alteration might precede altered *MBP* and *PLP1* expression. Determining the mechanisms that lead to an overexpression of *CNP* mRNA in MDD poses a promising field for future research.

Another aspect that has been investigated in WM is oxidative stress. Szebeni et al. measured levels of 8‐oxo‐2ʹ‐deoxyguanosine (8‐OXO) as a marker of oxidative stress in the anterior prefrontal cortex of deceased MDD patients. 8‐OXO levels were significantly elevated in Brodmann's Area 10 in the medial prefrontal cortex in deceased MDD patients compared to controls (Szebeni et al., 2017). Since many, but not all, MDD patients included in the study died of suicide, the authors further investigated whether suicide and oxidative stress show correlations. However, 8‐OXO levels were not significantly different in MDD patients who died from suicide compared to MDD patients who died of another reason, indicating that psychiatric illness itself is linked to higher levels of oxidative stress (Szebeni et al., 2017). Moreover, the study investigated the expression of the DNA repair enzymes poly‐ADP‐ribose polymerase 1 (PARP1) and oxoguanine glycosylase 1 (OGG1), which are both considered to be markers of oxidative stress in cells (Szebeni et al., 2017). A significantly higher expression of PARP1 and OGG1 in oligodendrocytes of MDD patients could be identified in the uncinate fasciculus and Brodmann's Area 10. Moreover, MDD patients exhibited a significantly increased *PARP1* expression in astrocytes in Brodmann's Area 10, while no group differences in the *OGG1* expression could be observed in these cells (Szebeni et al., 2017). Of note, neither chronic alcohol consumption nor smoking was found to be associated with differences in DNA oxidation levels, and no significant correlations between length of illness in MDD patients and DNA oxidation levels could be found (Szebeni et al., 2017). The authors hypothesize that oxidative damage might lead to elevated PARP1 activity, thus depleting cellular energy supplies and interfering with important functions of oligodendrocytes (Szebeni et al., 2017). A mechanism that has been proposed in this context is a pro‐inflammatory effect of poly‐ADP‐ribose, which is synthesized by PARP1. Once cleaved from proteins, poly‐ADP‐ribose has been found to trigger an inflammatory response in human and mouse macrophages, acting as an extracellular damage‐associated molecular pattern (Krukenberg et al., 2015). This mechanism could therefore mediate the process of DNA oxidation leading to neuroinflammation, which in turn has been repeatedly shown to be associated with depression, as reviewed by Kim et al. (2016). As chronic stress has been shown to cause significantly higher 8‐OXO levels in rats (Szebeni et al., 2017), DNA oxidation could represent an intermediate step between psychosocial stress and neuroinflammation.

In rats, Szebeni et al. also evaluated whether social defeat or unpredictable stressors cause alterations in DNA oxidation. It was determined that this double stress protocol was able to induce anhedonia and reduced social interaction (Szebeni et al., 2017). Moreover, a significant increase in DNA oxidation in WM, but not in grey matter, could be determined as well (Szebeni et al., 2017). These translational findings suggest that DNA oxidation in WM is specifically linked to depression and stress, and is unlikely to be influenced by common confounders such as alcohol consumption and smoking (Szebeni et al., 2017).

A further translational study investigated correlations between rodents and humans concerning the role of MicroRNA‐21 (MiR‐21) in MDD. Generally, microRNA plays an important role in gene regulation by binding protein‐coding mRNA strands and inhibiting their translation (Bushati & Cohen, 2007; Miguel‐Hidalgo et al., 2017; Valencia‐Sanchez et al., 2006). MiR‐21 has been mostly linked to carcinomas of the digestive system (Fu et al., 2011), as well as to glioblastomas (Møller et al., 2013), and has only recently been associated with depression, schizophrenia, and alcoholism (Miguel‐Hidalgo et al., 2017). MiR‐21 knockout mice were found to have a significantly higher PDGFR‐α staining in the corpus callosum compared to wild‐type mice, thus suggesting the presence of an increased number of oligodendrocyte progenitor cells (Miguel‐Hidalgo et al., 2017). Further staining against chondroitin sulfate proteoglycan 4, a marker for oligodendrocyte progenitor cells, revealed no significant group differences in the number of positive cells, so that the exact implications of the increased PDGFRα staining remain to be determined (Miguel‐Hidalgo et al., 2017). Nevertheless, the area fraction of MBP immunoreactive fibers in the anterior cingulate cortex of MiR‐21 knockout mice was significantly lower compared to wild‐type mice (Miguel‐Hidalgo et al., 2017). Meanwhile, in postmortem samples of human subjects, miR‐21 expression (determined by rt‐qPCR) in the orbitofrontal cortex was significantly lower in alcoholism, MDD, and comorbid alcoholism and MDD than in control subjects (Miguel‐Hidalgo et al., 2017). Moreover, a significant decrease of *OLIG1* and *glial fibrillary acidic protein* (*GFAP*) mRNA in the WM of the orbitofrontal cortex could be identified in MDD patients compared to controls. No such differences were found in alcoholism or patients suffering from comorbid alcoholism and MDD (Miguel‐Hidalgo et al., 2017). While double immunofluorescence staining determined that MiR‐21 is primarily expressed in mature oligodendrocytes, it cannot be ruled out that it could be present in other cells as well (Miguel‐Hidalgo et al., 2017). Therefore, the reduced MiR‐21 expression identified in MDD patients cannot indisputably be linked to oligodendrocytes.

The exact implications of alterations in MiR‐21 expression reported in this study are difficult to determine, as various factors other than microRNA control the transcription of genes. Whether reductions in MiR‐21 expression are a cause or a side effect of the alterations in the proteins linked to myelination remains to be determined. However, decreased GFAP and OLIG1 staining in MiR‐21 knockout mice might suggest a pathophysiological role of MiR‐21 in contributing to alterations determined in astrocyte and oligodendrocyte density.

Nodes and paranodes of Ranvier have been investigated concerning their role in WM alterations in MDD as well. Miyata et al. found that chronic stress leads to significantly narrower nodes and paranodes in the corpus callosum of mice (Miyata et al., 2016). The expression of the contactin‐associated protein (CASPR) was also investigated. This protein is found in the paranodal region of myelinated axons, between nodes containing Na^+^‐channels and the juxtaparanodal region, containing K^+^‐channels (Miyata et al., 2016). CASPR is believed to play a role in intracellular signaling as well as neuron–glia interaction and it can be utilized as a marker protein to identify nodes (Miyata et al., 2016). Staining against CASPR showed that areas of CASPR reactivity were significantly lower in stressed mice, as was the width of nodes, which was found to be reduced by 55% compared to control (Miyata et al., 2016). The expression of Kv1.1, a voltage‐gated potassium channel, was also investigated. It was found that areas of Kv1.1 immunoreactivity were smaller in chronically stressed mice than in control mice. Moreover, the distribution pattern of this channel was significantly more diffuse in stressed animals than in controls (Miyata et al., 2016). Furthermore, in control mice, CASPR and Kv1.1 were expressed in distinct locations, whereas in stressed mice, the distributions were overlapping in the paranode/juxtaparanode region (Miyata et al., 2016). The study also found evidence for chronic stress disrupting normal axon–myelin adhesion, represented by a downregulation of Neurofascin in chronically stressed mice (Miyata et al., 2016). Neurofascin is a cell adhesion molecule involved in synapse formation and neural development and its deficiency has been associated with disruptions of node/paranode complexes, as well as with reduced neural functionality (Zonta et al., 2008). In accordance with this, Cathomas et al. found that in mice exposed to chronic social stress, a reduction in the expression of genes for different ion channels could be identified. The mRNA expression of the sodium channel type IV beta protein (Scn4b) was strongly downregulated in the basolateral amygdala. Scn4b is a sodium channel subunit and as such, it is found in nodes and regulates the voltage dependence of sodium channels (Cathomas et al., 2019). Furthermore, the potassium channel subfamily K member 2 (Kcnk2) and the adenosine A2a receptor were found to be downregulated in the basolateral amygdala (Cathomas et al., 2019). Of these proteins, Kcnk2 has been associated with MDD, as distinct single‐nucleotide polymorphisms in this gene were found significantly more often in patients with MDD than in healthy subjects (Liou et al., 2009). Chronic stress also significantly decreased the Na+/K+ ATPase density and activity in the fiber tract of the corpus callosum (Miyata et al., 2016). The Na+/K+ ATPase requires energy in the form of ATP to function properly. A possibility that should be considered in this context is whether this lower activity could be caused by energy depletion due to DNA oxidation. To identify alterations on a genetic level, the authors used immunocytochemistry. Dexamethasone was applied to a cell culture containing mature oligodendrocytes and oligodendrocyte progenitor cells. The authors then investigated the serum and glucocorticoid‐regulated kinase 1 (SGK1) gene. SGK1 is a transcription factor controlling a myriad of cellular functions, including cell proliferation, apoptosis, and regulation of cell volume (Miyata et al., 2016). It has gained attention in neuropsychiatric research due to its ability to repress the transcription of the metabotropic glutamate receptors 3 and 5 (Miyata et al., 2016). The authors found that the expression of *SGK1* mRNA was significantly increased by dexamethasone stimulation, whereas the expression of metabotropic glutamate receptor 3 and −5 mRNA was significantly decreased (Miyata et al., 2016). This suggests a decreased oligodendrocyte activity following stress, which could lead to impaired interaction between mature oligodendrocytes and axons (Miyata et al., 2016). Moreover, chronic dexamethasone administration in the cell culture led to the formation of more complex and longer processes than in oligodendrocytes that were kept under control conditions (Miyata et al., 2016). Furthermore, the formation of myelin‐like sheath in dexamethasone‐treated cells decreased in comparison to controls (Miyata et al., 2016). These in vitro findings are in line with chronically stressed mice showing similar alterations in oligodendrocytes in vivo, as reported in the same study (Miyata et al., 2016).

Another gene that has been investigated regarding alterations in MDD is *CNP*. Mice that were heterozygous for this gene (*CNP^+/−^*) were used to test the effects of a mild loss of function of this protein. These mice showed a significant increase in microglia, infiltrating T‐lymphocytes as well as astrocytes in the corpus callosum, the striatum, and the anterior commissure (Hagemeyer et al., 2012). In *CNP^+/−^* mice, alterations became more pronounced with increasing age, showing an age‐dependent increase in neurodegeneration, detected by amyloid precursor protein staining (Hagemeyer et al., 2012). Also, an age‐dependent decrease in the expression of *CNP* mRNA was present in wild‐type and *CNP^+/−^* mice but was more prominent in heterozygous mice (Hagemeyer et al., 2012). Findings concerning altered behavior in *CNP^+/−^* mice could be confirmed in a study conducted by Cathomas et al. *CNP^+/−^* mice showed a more distinct activation of microglia, T‐lymphocytes, and astrocytes, as well as axonal swelling in both grey matter and WM (Cathomas et al., 2019). Taken together, *CNP^+/−^* mice seem to show a more pronounced inflammatory phenotype with progressing age as well as stronger axonal degeneration compared to wild‐type mice. Concerning the effects of *CNP^+/−^* on behavior, different studies utilized several tests, carried out on 24 months old mice. While no significant changes could be found in the open field test between *CNP^+/−^* and wild‐type mice (Hagemeyer et al., 2012), open arm visits in the elevated plus maze were significantly reduced in the *CNP^+/−^* group (Hagemeyer et al., 2012), meaning heterozygous mice showed normal motor activity and a mildly elevated anxiety profile. Also, *CNP^+/−^* mice showed reduced social interaction (Cathomas et al., 2019; Hagemeyer et al., 2012), loss of interest, higher floating time in the Morris water maze test, longer immobility time in the tail suspension test, and a catatonic state (Hagemeyer et al., 2012). The fact that the alterations in *CNP^+/−^* mice were found to be age related might indicate that heterozygosity for this gene poses a vulnerability factor for alterations, which, however, still requires a second hit (e.g., aging) to unfold its effects (Hagemeyer et al., 2012). This phenomenon is supported by human studies, which found that elderly patients with MDD are more likely to present with symptoms of catatonic depression, whereas these symptoms are absent in virtually all young patients (Hagemeyer et al., 2012). Cathomas et al. also studied the expression of oligodendrocyte‐related genes in mice following chronic stress. Gene expression was evaluated using rt‐PCR on samples obtained from the ventromedial prefrontal cortex, the basolateral amygdala, as well as the central nucleus of the amygdala. In the ventromedial prefrontal cortex, genes for MBP and Myelin‐associated Oligodendrocyte Basic Protein (MOBP) showed reduced mRNA expression, whereas such differences could not be detected in *MOG*, *MAG*, and *PLP1* mRNA (Cathomas et al., 2019). In the basolateral amygdala, the expression of genes coding for MBP, MOBP, and CNP1 were downregulated (Cathomas et al., 2019). The central nucleus of the amygdala demonstrated decreased mRNA expression of MBP and MOBP (Cathomas et al., 2019). Moreover, a 20% decrease in the oligodendrocyte population of the basolateral amygdala was identified (Cathomas et al., 2019).

Several further studies could confirm that stress and a depressive phenotype lead to WM alterations in rodents. In this context, a significant reduction of MBP in rats expressing a depressive‐like phenotype could be identified (Gao et al., 2017). Moreover, after being exposed to different stress protocols, rats showed a decrease in total WM volume (Chen et al., 2016; Gao et al., 2017; Xiao et al., 2018), a decrease in total length (Gao et al., 2017; Xiao et al., 2018), total volume (Xiao et al., 2018), and mean diameter of myelinated fibers (Gao et al., 2017; Xiao et al., 2018), as well as decreased total volume and thickness of myelin sheath. Moreover, shorter total capillary length, lower total capillary volume, and smaller total capillary surface area could be identified in rats after stress exposure (Chen et al., 2016).

An aspect that has recently gained attention is whether WM alterations can be reversed using exercise. In this context, running exercise daily for 4 weeks could be repeatedly shown to reduce depressive‐like behavior in a rat model of depression (Chen et al., 2016; Xiao et al., 2018). Moreover, rats that underwent a 4‐week running exercise protocol did not significantly differ from control animals concerning total WM volume (Chen et al., 2016; Xiao et al., 2018), the total length of myelinated axons, total volume of myelinated fibers, total volume, and thickness of myelin sheath (Xiao et al., 2018) as well as total capillary length in WM (Chen et al., 2016). It is important to note that in these experiments, exercise led to an absence of WM alterations despite being carried out after stress exposure. In contrast, antidepressants could until now only be shown to lead to an absence of WM alterations if applied simultaneously with stress (Abdel‐Wahab & Salama, 2011; Wang et al., 2014), but not if applied afterwards (Gao et al., 2019).

The results show that further research concerning the therapeutic and preventive properties of exercise and antidepressant medication is required to utilize these methods to their fullest capacities in the treatment of MDD.

## 5 DISCUSSION

In the present review, translational findings concerning WM alterations in depression have been presented. Based on these findings, altered diffusional metrics in interhemispheric tracts connecting frontal lobes could represent pathological alterations in brain circuits responsible for emotion regulation, thus contributing to a depressive phenotype.

Imaging studies provide strong evidence that inter‐ and intrahemispheric connectivity, as well as connectivity between cortical and subcortical regions, is altered in depression. A decrease in FA in the corpus callosum, the superior longitudinal fasciculus, the inferior fronto‐occipital fasciculus, the forceps major, as well as in the anterior limb of the internal capsule across species was evident. Concerning neurobiological implications of decreased FA, it has been repeatedly shown that this diffusion tensor imaging metric predominantly reflects myelin sheath integrity (Heckel et al., 2015; Kochunov et al., 2007, 2012; Lee et al., 2021). The most stable finding in treatment‐naïve patients with MDD was a disruption in the WM integrity of rostral regions of the corpus callosum (Guo et al., 2012; Sugimoto et al., 2018; Won et al., 2017; Yang et al., 2017). In terms of impaired interhemispheric connectivity, it is worth mentioning that the two hemispheres hold different functions and also show structural and functional asymmetries (Lai, 2019; Mundorf & Ocklenburg, 2021). For example, the left frontal lobe is more strongly involved in cognitive decision‐making and context‐related behavior, whereas the right frontal lobe is critical for tackling challenges posed by novel cognitive situations as well as for context‐independent behavior, thus highlighting the importance of interhemispheric communication between the left and right frontal lobes (Goldberg et al., 1994). Consequently, impaired interhemispheric connectivity can alter the communication between the left and right hemispheres or lead to atypical asymmetrical functioning causing significant impairments that are present in psychiatric patients (Mundorf, Peterburs, et al., 2021).

Despite representing a valuable development in MRI technology, no studies that conducted neurite orientation density and dispersion imaging, diffusion spectrum imaging, or myelin water imaging were included in the current review as the few existing studies did not meet the inclusion criteria. Studies utilizing these relatively novel metrics are scarce, especially in patients suffering from MDD. These metrics offer a promising future perspective for the identification of alterations in the brain of MDD patients. Especially as alterations in neurite orientation density and dispersion can already be identified in healthy participants with subclinical depression (Mundorf, Schmitz, et al., 2021).

Furthermore, imaging studies conducted on macaques exposed to early life stress could confirm aberrant myelination in the anterior limb of the internal capsule (Coplan et al., 2016), a region that is myelinated postnatally in macaques (Coplan et al., 2016) as well as in humans (Staudt et al., 2000). This finding provides further evidence for aberrant myelination in areas that are subject to maturation during early life in individuals subjected to chronic stress in this crucial phase of ontogenetic development. As the anterior limb of the internal capsule belongs to the structures showing postnatal maturation, aberrations of the myelination process of this tract could represent an important link between early life stress and a depressive phenotype. Moreover, rodent studies confirmed that both genetic alterations (van der Marel et al., 2013; Zalsman et al., 2017) as well as chronic stress (Kumar et al., 2014) were able to induce neuronal alterations comparable to findings in humans suffering from MDD.

Despite providing a valuable method to assess cerebral structures, limitations of diffusion tensor imaging should not be neglected. Considering that diffusion tensor imaging metrics assess the uniformity of the direction of water movement, crossing fibers, as well as kissing fibers can alter the diffusion of water molecules without having the anatomical correlate of aberrant connectivity and thus can interfere with the interpretation of altered diffusion properties (Pujol, 2015). While these phenomena might interfere with the results of MRI studies, reproducible findings of reduced FA in distinct brain areas point to the fact that alterations in diffusion tensor imaging metrics do not solely arise from artifacts. Moreover, there is widespread agreement about the fact that diffusion tensor imaging metrics represent an adequate tool to assess fiber integrity as well as myelination and thus, ultimately, connectivity (Beaulieu, 2002; Gosselin et al., 2009; Kochunov et al., 2012; Larvie & Fischl, 2016; Mädler et al., 2008). However, to reduce these potential confounders, researchers can use MRI tractography in humans to segment tracts such as the cingulum bundle. This tract has been delineated into five segments, each connecting different brain structures and being parts of distinct functional entities (Wu et al., 2016). This subdivision allows for a separate assessment of distinct parts of the cingulum bundle and can thus represent a field of future research to help clarify whether this tract belongs to the brain structures showing unambiguous alterations in MDD.

Findings from postmortem studies allow the unique possibility to determine pathological alterations underlying altered diffusion tensor imaging metrics. In humans, having experienced childhood abuse is associated with decreased total oligodendrocyte density in gyral WM (Lutz et al., 2017; Tanti et al., 2018), showing a shift toward a more mature phenotype of oligodendrocytes (Tanti et al., 2018). Interestingly, these alterations were present in victims of childhood abuse, but not in MDD patients without childhood abuse (Tanti et al., 2018). This suggests that alterations in the characteristics of oligodendrocytes might not directly be linked to MDD but rather represent a consequence of early life stress. Nevertheless, early life stress is considered a risk factor for psychopathological disorders (Carr et al., 2013; Mundorf & Freund, 2019; Mundorf, Kubitza, et al., 2021) and is associated with the development of MDD before the age of 18 years as confirmed in a recent meta‐analysis including 44,066 subjects (LeMoult et al., 2020). Consequently, the role of altered characteristics of oligodendrocytes following childhood abuse should be considered as a possible pathomechanism leading up to MDD. Therefore, analyzing MDD patients with or without childhood abuse separately might render more precise and stable results.

In this review, evidence for age‐dependent alterations in myelination in both neuroimaging and postmortem studies could be shown. Especially in MDD patients having experienced childhood abuse, results show more pronounced maturation of oligodendrocytes in years closer to the childhood abuse experience (Tanti et al., 2018). Since the total density of oligodendrocytes is decreased, while the density of mature oligodendrocytes was increased in these patients, a possible mechanism as a consequence of childhood abuse is demyelination. In support of this, Jiang et al. identified increased MOG and MAG serum levels, indicating demyelination, in depressive patients (Jiang et al., 2018). While typical demyelinating diseases such as multiple sclerosis present with different symptoms than MDD, these diseases show a strong association with depression, as reviewed by Siegert and Abernethy (2005). A possible overlap in the pathomechanism of multiple sclerosis and depression is therefore likely.

Not only demyelination but also insufficient myelin synthesis is a possible mechanism resulting in WM alterations. In this context, DNA oxidation has been suggested as a link between stress and aberrant myelination (Szebeni et al., 2017). Stress has been shown to cause DNA oxidation, which is known to deplete intracellular energy reserves (Szebeni et al., 2017). Energy depletion leads to intracellular alterations that inhibit the HMG‐CoA‐reductase, thus reducing the production of cholesterol, one of the most important building blocks of myelin (Saher et al., 2005). Further evidence in support of this hypothesis is neuroinflammation, a process that is a consequence of DNA oxidation and has also been identified in the context of MDD. DNA oxidation as a consequence of stress has been shown in mice, as well as in MDD patients (Szebeni et al., 2017). Childhood abuse, as a stressor, could cause DNA oxidation, which could then ultimately lead to decreased myelination. This hypothesis is in line with the fact that macaques exposed to early life stress show decreased myelination in postnatally maturing brain areas, but not in areas where the maturation is already completed prenatally (Coplan et al., 2016). Since frontal areas show postnatal maturation in humans as well (Staudt et al., 2000), this mechanism might also be present in MDD patients having suffered from childhood abuse. The results further support the proposal that the assessment of neuroimaging differences between MDD patients with and without childhood abuse could pose a promising field of future research. Further longitudinal studies in animal models of depression concerning age‐related neuroimaging correlates of WM could provide deeper insights.

The development of neuroimaging biomarkers of depression currently poses an important field of research (Lai, 2019; Mundorf et al., 2021; Mundorf & Ocklenburg, 2021). Identifying such biomarkers could ease the work of clinicians, providing a tool to distinguish between different psychiatric disorders. As presented in our review, many alterations in diffusion metrics are inconsistent and remain to be reliably replicated. One of these findings is FA reductions in frontal areas of the corpus callosum. So far, FA reductions in the genu of the corpus callosum are absent in several neurologic and psychiatric diseases, such as in unmedicated patients suffering from schizophrenia (Gasparotti et al., 2009) or multiple sclerosis (Hasan et al., 2005). However, this finding does not seem to be entirely specific to MDD, as it has been identified in migraine (Yuan et al., 2012) as well as in bipolar disorder (Wang et al., 2008). Moreover, one study has found that healthy adolescents at familial risk for unipolar depression exhibit decreased FA values in distinct brain regions, including the splenium of the corpus callosum (Huang et al., 2011). Since there is currently no unequivocal WM alteration that is specific to MDD, this neuroimaging method is not yet suitable to serve as a reliable biomarker for depression exclusively but rather as a marker of psychopathology. Further studies with more refined criteria are required to identify potential subgroups of patients expressing reproducible WM alterations.

To conclude, neuroimaging studies, as well as postmortem examinations, point to an important role of WM in MDD. Alterations in inter‐ and intrahemispheric communication might have a vast impact on cognitive and emotional behavior, thus leading to deficits observed in MDD patients. Preventing WM alterations early on might prove a promising step toward reducing symptom severity and disability. Animal models of depression show similar alterations and thus might help the study of potential pharmacological targets. Moreover, studies investigating longitudinal WM changes in animals could provide a valuable resource to assess the age dependence of WM alterations. A translational combination of these studies could provide important information to entirely unravel the mystery of WM alterations.

## 6 LIMITATIONS AND FUTURE OUTLOOK

Studies investigating WM alterations in the pathology of MDD hold great potential. However, some limitations and risks of bias have to be considered when interpreting the results.

A limitation of the current review is the fact that it does not provide a meta‐analysis of the conducted studies. This is not possible for several reasons. First, the number of studies available in each category is too small to allow for a statistically valid meta‐analysis. Moreover, not all included studies investigated the same anatomical areas, thus further reducing the number of studies available for a meta‐analysis of distinct regions. Therefore, this review gives an overview of the results of conducted studies but cannot provide a meta‐analysis of results.

Also, the present analysis runs the risk of containing selection bias, especially in the studies conducted on humans. MRI studies investigating only first‐episode, untreated MDD patients ensure comparability of the studies and exclude alterations caused by psychotherapy or pharmacotherapy, but do not represent the entirety of patients suffering from MDD. Nevertheless, the utilized criteria allow for standardized analysis of the impact of MDD on WM, while eliminating the most important confounders. Furthermore, investigation of brain tissue from postmortem donors bears the risk of selection bias as well. Most postmortem studies investigate suicidal MDD patients, thus resulting in the selection of only severe MDD cases. However, one also has to consider the availability of postmortem brain tissue, which provides a limited sample only. Weighing risks of bias against the availability of studies, the current approach provides the highest level of comparability of publications for a systematic review of different aspects of WM alterations in treatment‐naïve MDD patients.

Another limitation arises from the age span of patients included in the studies reviewed. FA values change for the life span of patients, increasing in adolescence, peaking at the age of 26–38 years, and showing a constant decline thereafter (Kochunov et al., 2012). After the age of 65, the FA decrease strongly accelerates, thus leading to a significant age‐dependent FA change (Kochunov et al., 2012). To minimize the impact of this confounder, studies examining patients under the age of 18 or above the age of 65 were excluded. This age range allows for an analysis of a collective with relatively age‐independent FA values while ensuring a sufficient number of patients is included. Additionally, potential sex differences are to consider, given the higher prevalence rates of MDD in women (Kuehner, 2017; Noble, 2005). However, in a comparably large study, Takao et al. conclude that sex differences in FA, which have been reported in some studies, do not reflect microscopic differences and are attributable to head size, rather than sex itself (Takao et al., 2014). Focusing on sex differences, especially on the role of sex differences in the context of microstructural WM alterations, could represent a promising field of future research to further develop the understanding of pathomechanisms of MDD.

Further confounders can be varying clinical parameters of patients. As previously mentioned, age of onset can alter FA, with early‐onset depression being accompanied by increased FA values, while late‐onset depression has been correlated with decreased FA (Cheng et al., 2014). Moreover, disease duration has been associated with decreased FA values in the corpus callosum (Kieseppä et al., 2010). Furthermore, treatment‐resistant MDD patients have been observed to exhibit lower FA values, specifically in the anterior limb of the internal capsule, as well as the corpus callosum and the external capsule when compared with treatment‐responsive MDD patients (Guo et al., 2012). These results show that even among MDD patients, variances due to different clinical features can be present and can influence diffusion metrics. To minimize the effect of these possible confounders, we excluded studies that investigated patients who have already received treatment in the form of antidepressant medication or psychotherapy, thus limiting the extent to which these features could alter results and ensuring that the patient collective is as homogenous as possible. Regarding the included studies on animal models, the timepoint of investigation, for example, time until testing after stress exposure, varies across studies. Therefore, timing of analysis could be another influential factor leading to different results across studies.

Still, neuroimaging studies and postmortem examinations consistently point to an important role of WM alterations in the pathology of MDD. Further studies are needed to truly disentangle the mystery of WM alterations in MDD. Future studies should include more participants with a more differentiated clinical history assessment. Utilizing standardized diffusion tensor imaging metrics could further help yield data for the conduction of meta‐analyses. Moreover, providing age‐adjusted FA values, for example, through assessment of the FA in the spinal cord, could allow for complete elimination of age as a confounder.

### PEER REVIEW

The peer review history for this article is available at https://publons.com/publon/10.1002/brb3.2629.

## Supporting information

Supplementary Table 1‐ Imaging Studies in MDD Patients

Supplementary Table 2‐ Imaging Studies in Animal Models of MDD

Supplementary Table 3‐ Post‐Mortem Studies in MDD Patients

Supplementary Table 4‐ Post‐Mortem Studies in Animal Models of MDD

## Data Availability

The data that support the findings of this study are available from the corresponding author upon reasonable request.
